# On the Diffiiculties and Advantages of Catheterism of the Air-Passages of the Chest

**Published:** 1860-03

**Authors:** Horace Green


### Miscellaneous.

*On the Difficulties and Advantages of Catheterism of the Air-Passages of the Chest.* By HORACE GREEN, M. D., LL. D., &c.

(Read before the Medico-Chirurgical College, Dec., 22, 1859.)

In December, 1854, I read a paper before the Academy of Medicine of New York, "On the Injection of the Bronchial Tubes and Tubercular Cavities of the Lungs;" and subsequently, namely, in March, 1856, I published in the *American Medical Monthly* a detailed report, containing a statistical table of one hundred and six cases of pulmonary and bronchial diseases, treated by means of catheterism of the air-passages, conjoined with appropriate general remedies.

Still continuing, to some extent, this plan of topical treatment in thoriacic disease, I have since had opportunities to confirm the truth of some of my early observations; and, what is of equal importance, to correct other views which later experience and more extended observations have shown to have been erroneous conclusions. It is to record and announce these recognized errors, and to point out some of the difficulties, as well as the advantages that attend this plan of treatment, that I bring, at this time, the subject of Topical Medication before the College.

I propose briefly to consider the following questions:

1. Can the operation of catheterism of the air-passages be performed with certainty and facility?

2. What are the difficulties and dangers of the operation?

3. What advantages are to be derived from this method of treatment?

1. With regard to the first inquiry—the possible practicability of the operation? On this point it will not be necessary long to dwell. As very few of the profession, at the present day, will deny its performance, under favorable circumstances, I

shall only refer to the opinion of a few members of the profession, from among many of those who have considered this question.

At the discussion that followed the reading of my paper, to which I have alluded, on bronchial injections, before the Academy of Medicine, several years ago, it was remarked by a distinguished member of that body, who denied the practicability of the operation, that "the Academy must not decide this question until we had heard from Europe on the subject, as the profession there would act without prejudice or partiality."

Already, testimony has come to us from eminent men of the profession, in Great Britain, France, and Germany, that this operation of injecting the bronchi has by them been successfully performed.

Prof. J. Hughes Bennett, of Edinburgh, in his work, "*Clinical Lectures on Medicine*," says: "I have now introduced the catheter publicly in the clinical wards of the Royal Infirmary, in several patients affected with phthisis, in various stages, in laryngitis, and in chronic bronchitis, with severe paroxysms, of asthma. * * * I have been surprised at the circumstance of the injections not being followed by the slightest irritation whatever, but rather by a pleasant feeling of warmth in the chest, (some have experienced a sensation of coolness,) followed by ease to the cough, and a check for a time to all expectoration." "These facts are made known to the profession," Dr. Bennett declares, "with a view of recommending a practice which, if judiciously employed, may form a new era in the treatment of pulmonary diseases."*

In Paris, Prof. Trosseau, Loiseau, Blondeau, and others, have succeeded in injecting the air-passages, in various diseases of these parts. It has been employed in early phthisis, by M. Trosseau, as well as in diphtherite, in which latter disease it was attended with complete success.

It has been still more extensively employed by Loiseau, in the treatment of both diphtheritis and croup. The method of Loiseau is thus described by Trosseau, who was appointed

*See Clinical Lectures on Medicine, p. 609.

by the Imperial Academy of Medicine, of Paris, to report upon his plan of treatment: "With the extremity of the forefinger," says M. Trosseau, "he (Loiseau) depresses the tongue, seizes the epiglottis, raises it, and presses the end of the finger between the arytino-epiglottic folds. There is then nothing more easy than to make the end of the tube glide over the finger. The air which escapes through the exterior extremity of the tube proves that it has really entered into the larynx. Through this tube, serving as a conductor, a caustic, the nitrate of silver, for example, or any other medicated substance, may be carried."

In the discussion which took place at the time, before the Academy, on this subject, M. Depaul said, "The process of catheterism of the larynx, as proposed by Dr. Green, was declared by some as being very difficult, even upon the cadaver; but I maintain," said he, "that nothing is easier than this catheterism for those who have performed it a certain number of times." Still more recently than this, comes to us the testimony of Prof. Greisenger, of Germany, as reported in the *Deutsche Klinik*, and in the *Gazette Hebdomadaire.*— Prof. Greisenger has been able, as he affirms, to introduce medications of nitrate of silver solution into the air-passages. In regard to the practicability and danger of this operation, Prof. G. says: "For us, after the experiments we have made, we can affirm that these fears are illusory, and that the different parts of the operation can be performed with a rigorous exactitude." And, finally, we have the testimony of the Committee, appointed by the New York Academy of Medicine, to inquire into the truth of the performance of this operation; for they affirm, in their report, made to the Academy, that of the thirty-two patients upon whom the attempt was made to inject the bronchi, the operation was performed in *eleven* cases successfully, and to the entire satisfaction of the committee. It must, therefore, be concluded that the "operation of catheterism of the air-passages," under appropriate circumstances, can be positively performed.

Notwithstanding this operation is being daily performed at the present time, yet it is not always accomplished with certainty and facility. Nature has so guarded the opening into ærien passages that catheterism of the bronchi is an operation that will be found difficult to accomplish. In many cases, I am confident, the tube passes over the glottic aperture, and enters the œsophagus, even when the operator feels quite certain that it has been introduced into the larynx. In my own practice I have found myself deceived, not unfrequently, especially in the first years of my experience in this mode of treatment. At first I believed the instrument to have taken the right course, but afterwards ascertained, in many instances, that it had entered the œsophagus.

2. What, then, are the difficulties that oppose themselves to the facile performance of this operation, and what the dangers?

The *epiglottis* does not of itself close entirely the aperture of the glottis. This cartilage being placed between the entrance of the larynx and base of the tongue, is pressed downward by the abasement of the latter, in the act of deglutition, and being moulded upon, only partially closes the glottis. It is not, therefore, correct to state, as many anatomists do, that the epiglottis "closes completely, of itself, the opening of the larynx," in deglutition.

The arytenoid muscles are the especial *constrictors* of the glottis. These muscles (as Longet has demonstrated) receive filaments from the recurrent nerve. Covering the lips of the glottis is a narrow zone of exquisitely sensitive mucous membrane, which receives its nervous filaments from the internal branch of the superior laryngeal nerve. These two nerves, the one supplying the constrictors, and the other this strip of mucous membrane, communicate freely with each other, but they have no connection whatever with the epiglottis. The irritation of this body, therefore, will have no effect upon either the motive or sentient nerves peculiar to the larynx.— This is important to remember, namely: that the epiglottis, in its normal state, is an organ nearly insensible; but when the least irritation of that sensitive portion of the mucous membrane which covers the supra-glottic space occurs, this irritation is quickly communicated to the constrictor muscles, through filaments of the recurrent and laryngeal nerves, and the aperture of the glottis is as quickly shut up. When it is desirable, therefore, to medicate the ærien passages in disease of these parts, it is necessary, as all are aware, to educate the glottic aperture, by repeated cauterizations of this opening. For if, under ordinary circumstances, the attempt be made to pass the sound, or probang, into the larynx before the exquisitely normal sensitiveness of this point of membrane be partially subdued, it will probably prove abortive; or, if successful, and the instrument be made to pass the supra-glottic guard, a violent spasmodic action, not only of the constrictors, but all the other muscles of the larynx, will occur, followed, often, by great irritation of the parts, and a suffocative cough; and if, under these circumstances, the operator persist in finishing the operation, by injecting a solution of the nitrate of silver into the bronchi, the irritation and cough are both greatly increased, and in some instances inflammation of the bronchial and pulmonary tissue have been awakened, apparently by these combined disturbing causes. This condition, as the result of these causes, may be illustrated by the following case:

Mrs. F., a widow lady, aged 35, recently returned from California, came under my care July 31, 1858; she was in the second stage of tubercular consumption. Auscultation revealed tubercles, with softening in the right lung. The disease of the lungs had been preceded by follicular laryngitis for many months. The right tonsil, which was still ulcerated, was nearly destroyed, and the pharynx was granular from the diseased and enlarged follicles.

She was placed under general treatment, ordinarily adopted in such cases, together with the application of a solution of nitrate of silver to the throat.

This treatment was continued until the 13th of August, when the parts were thought sufficiently prepared to allow the introduction of the injecting tube. On this day I introduced without any difficulty, the tube, and injected a drachm of the nitrate of silver solution into the right bronchus. No irritation followed the operation. As is the case almost invariably, after injections in either pulmonary or bronchial diseases, the cough and expectoration were considerably diminished for several days after this operation. With intermediate cauterizations with the sponge probang, the bronchial injection was employed on the 16th, the 20th, and the 24th, with similar beneficial results with the first operation, the patient continuing constantly to improve. On the 26th of the month, in attempting to use the tube, the throat of the patient was found to be unusually sensitive, and it was with some difficulty that the instrument was introduced into the larynx. It was passed, however, into the trachea, in precisely the same way that it had been done on former occasions. A spasm of the glottis immediately succeeded its introduction, and instead of withdrawing it at once, as should have been done, I proceeded to finish the operation, and injected a drachm of the solution (15 grains to the ounce) into the bronchi. By the time the operation was completed, the whole chest seemed thrown into a violent spasmodic action; a convulsive cough, with dyspnœa, followed, which continued during several hours, but was finally somewhat relieved by the use of chloroform, and the administration of anodynes. The cough and dyspnœa, however, with increased expectoration, and pleuritic pains, continued for several days; and, although the patient became in the course of a week quite comfortable again, under general treatment, yet she never entirely recovered the favorable state she was in before the occurrence of the spasm. As the patient and friends were greatly opposed to any further *topical* treatment, it was never afterwards employed. The pulmonary symptoms increased, the disease progressed, as usual in such cases, and the patient died on the 10th of October, about two months after the last employment of the tube.

*Remarks.*—The above was a well-marked instance of tubercular disease of the lungs, following a long-continued case of folliculitis; one of those cases, in short, a great number of which in their early stage, in the hands of other practitioners, as well as in my own, have been, and are, successfully treated by topical medication, conjoined with general remedies; and although a *cure* in this case could not, probably, have been effected, yet, from the favorable progress made before the operation on the 26th, I am confident in the belief that the life of the patient would have been prolonged by the treatment, if it had not been for this untoward occurrence.

A case, similar to the one I have related, came under the observation of my assistant, Dr. Richards. Not having been present when the operation was performed by Dr. R., I take his account of the case.

The patient, Mr. D. M., had been long under treatment for obstinate chronic bronchitis. Topical medication, by means of the tube and sponge-probang, had been repeatedly employed and the patient had been greatly benefitted by the treatment. Mr. M. is the same patient whose case is mentioned by the Committee of the Academy of Medicine in their report, on Bronchial Injections. His case is No. 30; and the Commission thus speak of the success of the operation, as then performed in their presence; "The tube," say the Committee, "was passed without much strangling; the air was freely expelled through the tube. An injection of two or three drachms of a solution of the nitrate of silver, of the strength of thirty or forty grains to the ounce, was then thrown in.—All present were satisfied that the experiment was successful." In this instance, as the report affirms, no irritation followed the operation, nor had any irritation attended any previous operations. But on a subsequent occasion, namely, on the 20th of May, 1856, he called to have this tubing operation repeated. Dr. Richards, being in attendance that morning, introduced the tube in the same manner as it had been done both by Dr. R. and myself, on many former occasions. At this time, however, a spasm, from some cause, was immediately induced; Dr. R. did not withdraw the instrument, but proceeded to inject, as at other times. By the time the operation was finished, the muscles of the throat and chest were violently convulsed, and this was followed by a suffocative cough and profuse expectoration. This irritation, increased cough, and expectoration lasted during several days; but it finally subsided, and the patient ultimately regained a good degree of health.

I have before stated that Prof. Bennett has employed bronchial injections in the treatment of pulmonic diseases. In the *Edinburgh Medical Journal*, and in his work, recently published, on "Clinical Medicine," he has reported some most interesting cases, in which this method of treatment was employed.

Since the publication of the above work, by Prof. Bennett, I have been favored with a letter from him, on the subject of bronchial injections, in which, among other things, he alludes to the occurrence of an accident, in his own practice, similar to those whose history has been given. He writes: "A gentleman, in the last stage of phthisis, with cavities in both lungs, and tubercles very generally distributed among them, after long treatment with the probang, allowed me to inject the bronchi. I did so, and he was immediately seized with the most violent dyspnœa. I thought he would have died in my study. It continued several days, and then gradually declined. After five weeks confinement to bed, he was restored to the same condition he was in formerly. This was six months ago. My opinion is, that he made a too violent effort to hold his breath and retain the catheter, and either ruptured an emphysematus portion of the lung, or caused a small abscess to break, as the operation was followed by abundant purulent expectoration."

In a letter which I received during the present year, from the distinguished professor of Clinical Medicine in Paris, M. Trousseau, he, among other interesting statements made on topical medication, mentions the occurrence of an accident in his practice, from the use of nitrate of silver solution, under circumstances different from any that have come under my own observation. He remarks: "I often cauterize the interior of the larynx. I sometimes, but rarely, use a hollow caustic holder like that of Dr. Loiseau's, and I have also injected into the trachea solutions of nitrate of silver and sulphate of copper. This practice, in my hands, has never been attended by any danger, and I have never heard that Dr. Loiseau has had any accident to deplore. * * * I have introduced caustic solutions very frequently into the trachea and bronchial tubes, after tracheotomy, in case of croup. For six years I

never operated for tracheotomy without injecting caustic solution." "Once this practice," continues M. Trosseau, "in my hands, caused the immediate death of a child. The case was as follows: I had operated on a child two and a half years old; he breathed very well. I dropped into the trachea ten or fifteen drops of a solution of nitrate of silver; a coagulation of thickened mucus, which was in the principal bronchi, immediately followed, and the child died, strangled, in less than a minute." "An accident of this kind," he adds, "can never happen if a sponge, moderately wet with the caustic solution, be used; and with the instrument which you use, a model of which you have sent to me, I cannot see how an accident can occur to the lungs."

It would also seem impossible that this accident, to which Prof. Trosseau alludes, could have resulted from the cause to which he refers it. He had used it frequently before in the same manner, during a period of six years, without the occurrence of any such accident.

During the last winter, it will be remembered that many severe cases of membranous croup and diphtheritic inflammation occurred in some of our larger cities. This was the case particularly in Boston, Mass., in which city the physicians have reported some almost hopeless cases that were saved through the combined measures of tracheotomy, followed by repeated injections of a solution of nitrate of silver, through the opening into the trachea and bronchi. In one instance, as reported in the *Boston Medical and Surgical Journal*, in the case of a child, aged four and a half years, Dr. Gay, assist-by Drs. Bowditch and Perry, "injected through the artificial opening into the trachea, *every four hours*, about one-third of a teaspoonful of the solution of nitrate of silver, of the strength of 20 grains to the ounce of water." This treatment was continued through several successive days and nights, and resulted in the complete recovery of the patient. It would seem, therefore, that in the case reported by M. Trosseau, the patient must have died from some other cause than the one mentioned, namely: "ten or fifteen drops of a solution of nitrate of silver into the trachea."

Nor is there any need of the occurrence of any accident from the employment of catheterism of the bronchi, if proper cautions are adopted; for, with our present knowledge and experience in the use of this measure, it is one, we maintain, that may be employed with as much safety as any of our other remedial agents.

To the precautionary measures necessary to be adopted in topical medication I shall refer, after alluding to another danger.

When the attempt was first made to inject the trachea and bronchi, it must be remembered that there were no precedents, no recorded cases, in which this practice had been adopted, to which we could refer, for guiding us in regard to the strength of the remedies, or to the amount of medicaments that could with safety be injected; consequently, it became necessary to proceed with much caution, in the inauguration of this practice. Fortunately, those persons upon whom the attempt was first made to employ this method of treatment, were among those patients who for a long time had been under treatment for laryngeal and bronchial diseases; to whose larynges the sponge-probang had been frequently, and for a long time, applied; consequently they were particularly well prepared for the introduction of the injecting tube, and for the employment of the injections; and it was for these reasons that bronchial injections, in the first instances in which they were employed, were better borne, and were accomplished with more facility, than they have been in most instances since. At any rate, I soon found that in recent cases, I had more difficulty in effecting the introduction of the tube, and that it was necessary to employ, at first, a *very mild* solution, which could be subsequently increased in strength. The following case will illustrate one of the difficulties to which I refer:

In September, 1854, Miss H., a young lady of this city, was recommended to my care, by her friend and physician, Dr. C——, for the treatment of a bronchial affection. The ordinary signs of bronchitis were very marked. Topical applications, of the nitrate of silver solution, were made to the glottis and larynx, and the general remedies, ordinarily recommended in such cases, were administered. This treatment was continued several weeks without producing any decidedly beneficial effect upon the patient. About this time I saw the patient on several occasions, in consultation with the physician who had recommended her to my care. He advised a further perseverance in the plan of treatment, but suggested the employment of catheterism of the bronchi, (an operation he had seen performed, in similar cases, several times upon my patients) if the present measures, after a further trial, should be successful. But her disease continued to resist the influence of those measures which had proved quite successful in the management of other, apparently similar, cases. On the 7th of November, therefore, the bronchial tube was, with some difficulty, introduced, and nearly a drachm of the solution injected into the bronchi. An unusual amount of irritation followed this operation.

The introduction of the tube induced a spasm of the glottis; the patient coughed severely, and complained, while she remained in my office, of pain in the larynx and bronchi.—She, however, left soon after the operation, for her house, in the upper part of the city, but did not return for any further treatment. The subsequent history of her case was obtained afterwards, from herselt and her mother.

The cough and bronchial irritation continuing after her return home, the patient and her friends became alarmed, and called in their ordinary medical attendant, who, in turn, called in a consulting physician, but both concluded to do nothing, for the irritation gradually subsided, and, along with it, the alarm of the patient and her friends; and, still better, the cough and bronchial disease, which had so long and so obstinately resisted other measures, entirely disappeared, and the young lady has continued in good health, up to the present time.

Spasms of the glottis will, as I have before stated, occasionally occur, caused by the irritation of the supra-glottic space, in the introduction of the tube, although great pains may have been taken to prepare the parts by previous training. In this case, I at first attributed the spasm and subsequent cough and dyspnœa to irritation, produced at the glottic opening.—

But from some observations and experiments which I have since made, I am fully satisfied that the disturbance in this instance, and probably in the case mentioned by Dr. Bennett, as well as in some others, similarly affected, was caused by the employment, at first, of a solution of too great strength.

I have recently instituted some interesting experiments upon animals, (the cat and dog,) in order to ascertain how strong a solution of nitrate of silver can be borne, when injected into the trachea and bronchi. I experimented upon these different animals, but found the results the same, under similar circumstances, in both the cat and dog. But I will detain the College with the history of only one case.

A young dog, eight months old, weight fifty pounds, was treated by bronchial injections. His jaws were opened by an assistant; a cord being placed around his tongue, it was readily drawn out of his mouth, when the epiglottis, and the opening of the glottis, were seen without any difficulty. I passed the tube quite readily into the larynx, and carried it down eight inches, into the trachea. Here it was allowed to remain several minutes, without producing the least disturbance, while the respired air passed freely through the tube. After a time I injected a small amount of a weak solution of the nitrate of silver through the tube into the lungs of the animals; but, as he did not seem to be at all effected by this, I soon after threw in half an ounce of a solution of the strength of fifteen grains to the ounce. After being released, he commenced playing about as usual, without showing a symptom of any disturbance whatever. The next day he appeared perfectly well, and was as playful as ever. At 5 o'clock P. M. on the following day, I again introduced the tube into the dog's larynx, and conveying it down, nearly the whole length of his trachea, but not below the tracheal bifurcation, I injected into the bronchi the ounce syringe full of a strong solution of the nitrate of silver, of the strength of thirty grains to the ounce of water. This amount, in proportion to the weight of the animal, would be equivalent to three ounces of the solution of this strength to an adult. The respiration of the animal was not impeded at the time, nor did any signs of suffocation follow immediately this operation of injecting so large an amount of fluid into the air-passages. The dog, for a time, ran about as usual. At 7 o'clock, two hours after the operation, I visited him at his kennel, and calling him out, found him with tail hanging down, eyes dull, and breathing with some difficulty, and uttering occasionally a short cough. On listening to his sides, moist, bronchial, and crepitant rales were heard throughout both lungs. He was allowed to lie down in his kennel. At 10 o'clock I went to him again, when I found that all these symptoms had greatly increased; the dyspnœa was quite difficult, and the dog was disinclined to move about. He died during the night.

I examined the lungs the next day; the bronchial mucous membrane was highly inflamed. Both lungs were inflamed, and gorged with blood; and bloody and frothy mucus blocked up the bronchial tubes. The animal died, therefore, of inflammation of the lungs and bronchi, superinduced by the large and strong injection of a solution of nitrate of silver into the bronchi.

*Remarks.*—It is evident, then, that nitrate of silver may be used of that strength, and to that amount, in bronchial injections, as to prove fatal to animal life. So, also, may the too frequent use of all or of any of the potent remedies destroy life.

3d. In relation, then, to the third inquiry, "What advantages are to be derived from this method of treatment?" I reply: bronchial injections of a solution of nitrate of silver, when judiciously employed, have proved to be, and will continue, I believe, to be a valuable therapeutic means in thoracic disease.

In the commencement of this paper, I referred to the detailed report which was published by me, two or three years ago—a report containing a statistical table of one hundred and six cases of pulmonary and bronchial diseases, treated by means of catheterism of the air-passages, conjoined with appropriate general remedies. The following is the brief analysis given at the conclusion of the report of the above cases: "If we analyze the *one hundred and six cases*, reported in the table, it will be found that *seventy-one* of the sum-total have been recorded as cases of *advanced phthisis*—cases in which tubercular cavities were recognized, in one or both lungs; and *thirty-nine* cases of *early phthisis*. Of the first division—advanced phthisis—*fourteen* have since died. *Twenty-five* were more or less improved; their lives, apparently, being prolonged by this means of medication. *Seven* only of the thirty-two cases of advanced phthisis were not benefited by the injections. Of the *thirty-nine* cases of *incipient tuberculosis*, *twelve* of this division have apparently recovered. *Five* more of this number are now, or were, at the last report, in the enjoyment of a good degree of health. These five cases were classed by my assistant, Dr. Richards, with the twelve recoveries; making *seventeen*, in all, of the thirteen cases of early tuberculosis which have apparently recovered.

"Of the remaining *twenty-two* cases, many of whom are still under treatment, *seventeen* have been greatly improved by topical medication; *three* more have been moderately benefited; while *three* only have failed to obtain any advantage from the local measures which have been adopted.

"Of the *twenty-eight* cases of bronchitis, *sixteen* have been dismissed, cured, or so much improved as to require no further treatment. All the others have been greatly benefited.*"

This method of treatment, in this class of diseases, has been continued, more or less, since the report to which I have referred was made; and such has been the amount of success which has continued to attend this plan of treatment up to the present time, I am now ready to affirm, after an experience of many years, in a field of observation unusually large, that, *if I was required to relinquish all other known therapeutic measures or topical medication in the treatment of thoracic diseases, I should choose the latter, with hygienic means alone, in preference to the entire class of remedies ordinarily employed in the treatment of these diseases.* But I shall now refer briefly to the opinion of other physicians as to the value of this mode of treatment.

* See published "Report of One Hundred and Six Cases of Pulmonary Diseases, treated by Bronchial Injections," &c., pp. 34–5.

In chronic bronchitis, in asthma, and in early tuberclosis, cauterization of the air-passages has been found to be a most valuable and efficient remedy. As I have stated, topical medication, in the treatment of thoracic diseases, has been continued in my hands since the publication of the "Report of the One Hundred and Six Cases" to which reference has been made. During this period of three or four years, large numbers of patients, affected with chronic laryngeal and bronchial diseases, with asthma, and with tubercular phthisis, have been treated, and the success which has continued to attend this practice has served to increase greatly my confidence in this measure, as a therapeutic agent. I shall, however, omit a detail of any of these cases coming under my own observation, and only refer briefly to the opinion of other physicians on the value of this mode of treatment.

At a meeting of the French Academy of Medicine, subsequent to the reading of M. Loiseau's paper on catheterism of the Larynx in Disease, a very favorable report on the management of some of the diseases of the air-passages by this method was adopted; the commission making the report declaring that catheterism of the air-passages in the treatment of diphtheritic inflammation and other kindred affections, is not only practicable, but is of great utility.* "I believe this method," said M. Velpeau, "to be a good one. While diphtheritis is at the opening of the air-passages, it is curable, and M. Loiseau has ascertained that it is not difficult to carry medications into the larynx."†

"As a therapeutic means," says the editor of the *Gazette Medicale de Paris*, "it merits a more serious attention. What is the relation of cauterization to croup? It is a powerful, energetic means, *the only one, which, up to this time, has really succeeded.* When the disease is limited to the upper part of the air-passages, we cauterize, and all practitioners agree that this means is truly of great benefit. What is laryngeal cauterization other than carrying beyond the limits of ordinary cauterization, a remedy recognized as good, efficacious, not only against the essence of the disease itself, but

* See Union Medicale, Aug., 1857. † Ibid.

also against the pathological secretion?" And the learned editor of the *Gazette Hebdomadaire*, after calling attention to what had been done in America in the treatment of croup by cauterization, adds: "These experiments should be repeated by us, with that attention which the authority and the honorable position of our American confreres command. M. Loiseau, anticipated, as it is seen, in every particular, has given us, however, a useful example, and his merit will still be great if he succeeds in introducing into use a practice worthy of more attention than it has yet received."*

During the last year, the *Gaz. Hebdomadaire*, and other French journals, have contained the histories of several severe cases of diphtheria, which, under the care of Loiseau, Trosseau, Gros, and other physicians of Paris, were successfully treated, by catheterism of the larynx. In alluding to one case reported by M. Gros, where the diphtheritic inflammation had extended deeply into the air-tubes, threatening immediate suffocation, but which was permanently cured by injections into the larynx, the editor of the *Gaz. Hebdomadaire* says:

"This fact has an important practical signification, and speaks loudly in favor of the advantages which may be derived from catheterism of the air-passages, and from topical applications, carried by this measure directly into the larynx and trachea."†

Indeed, M. Trosseau has quite recently expressed, before the French Academy, his want of confidence in all the ordinary violent remedies in the treatment of croup, such as severe vomiting, blisters, leeches, etc., declaring his belief that we must place our main dependence upon direct catheterism, or cauterization of the air passages, followed, if this measure is unsuccessful, by tracheotomy.

In Dr. Hughes Bennett's work, to which I have already alluded, he has devoted a chapter to the consideration of "Injections of the Bronchi in Pulmonary diseases." He remarks, "Whilst tuberculosis is at first a constitutional disease, its localization in any part reacts more or less on the general

* Ut supra, Aug,. 1857.

† Gazette Hebdomadaire, Sept., 1859, p. 660.

health; and the opinion I have long entertained, that any means which could enable the physician to act directly on the tissue of the lung or inflamed bronchi, would assist his efforts at cure, at once led me to take a favorable view of this new mode of treatment. The nitrate of silver ought to act as beneficially on the mucous membrane of the trachea and bronchi as on that of any other hollow viscus, and we have seen previously that the remedy may be applied to the tracheal mucous membrane, by means of an artificial opening, not only without injury, but with decided benefit." He further adds, "Without entering into minute particulars, I have only to say that I have confirmed the statements made by Dr. Horace Green."

The cases in which Dr. Bennett employed this method of treatment, as he states in his work, were, patients "affected with phthisis in various stages, with laryngitis, and in chronic bronchitis, with severe paroxysms of asthma. In other cases in which I attempted to pass the tube, it was found to be impossible; in some because the epiglottis could not be fairly exposed, and in others on account of the irritability of the fauces, and too ready excitation of cough from pressure of the spatula."*

This, then, is only a part of what has been done in France, Germany, England, and Scotland, in the employment of topical medication in disease. In some of these countries, far more extensive observations on this mode of treatment have been made than in our own country; certainly, more than in our own city! But I shall not stop here to compare the careful inquiries, the scientific observations made and the frankness and candor exhibited, by the profession of other countries, on this subject; with the course pursued by many of my "American confreres;" nor, especially, with the *non-committalism* of the New York Academy of Medicine, before which body this matter of catheterism was first brought; and whose report on this subject has slept for five years, unmolested, on their table!

If necessary, I could give the opinion of many other practi-

* Clinical Lectures, &c., p. 609.

tioners, in Europe and America, who have tested topical medication, in the treatment of diseases of the air-passages, and who profess to have derived signal advantage from this therapeutical measure.

I will only refer to some favorable testimony from some parts of our own country. During the last year, as it was remarked on a former page, croup and diphtheria were more than ordinarily prevalent in some of our larger cities. This was the case particularly in Boston; and here, many very severe cases of diphtheria occurred, and some almost hopeless cases were saved by cauterizations of the larynx; and others, by tracheotomy; followed by repeated injections of a solution of nitrate of silver, through the opening, into the trachea and bronchi.

In a report of some most interesting cases of the disease, read before the Boston Society for "Medical Improvement," and subsequently published in the *Boston Med. and Sur. Journal*, Dr. Gay says, "After tracheotomy, and the insertion of the tube, the injection of a solution of nit. argent. through the tube, into the trachea and bronchi, is our strongest dependence and most of the other measures are mere auxiliaries." "In seven cases decided membranous croup," says Dr. Gay, "in which these combined measures were employed, and in which the membrane was expelled through the tube, there have been *five recoveries*, and *two deaths*." Many other severe cases were successfully treated by cauterizations of the larynx and trachea, employed before the operation of tracheotomy became imperative.

I shall close this paper by describing the method I employ in practicing catheterism of the bronchi. I have received let ters from many medical men, requesting me to give them an account of the manner of performing the operation, and a description of the instruments employed. As it has been, and is, impossible for me to comply with all these individual requests, I cannot do better than to reproduce the directions I sent to Prof. J. Hughes Bennett, who several years ago wrote to me, desiring me to send him a description of the operation, and a set of the instruments I employed. My reply is published at length in Prof. Bennett's recent volume of "Clinical Lectures," from which I shall extract.

"I would, with pleasure, send you the instruments I employ, but they are simple, and may be obtained at any surgical instrument maker's shop. They consist of an ordinary flexible, or gum catheter, and a small silver, or glass syringe.—The catheter is Hutchings' gum-elastic catheter, (No. 11 or 12) which is 12½ inches in length; and, as the distance from the incisor teeth to the tracheal bifurcation is, ordinarily, in the adult, about eight inches; if this instrument is introduced so as to leave only two inches of the catheter projecting from the mouth, its lower extremity must, of course, (if it enter the trachea) reach into one or the other of its divisions. I first prepare my patients by making applications, with the sponge probang, and nitrate of silver solution, for a period of one or two weeks, to the opening of the glottis and the larynx, until the sensibility of the parts is greatly diminished. Then, having the tube slightly bent, I dip the instrument in cold water, (which serves to stiffen it for a moment, and obviates the necessity of using a wire,) and with the patient's head thrown well back, and the tongue depressed, I place the bent extremity of the instrument on the laryngeal face of the epiglottis, and gliding it quickly through the rima glottidis, carry it down to, or below, the bifurcation, as the case may require. It is necessary that the patient continue to respire, and the instrument is most readily passed during the act of inspiration. The tube being introduced, the point of the syringe is inserted into its opening, and the solution injected. This latter part of the operation must be done as quickly as possible, or a spasm of the glottis is likely to occur. Indeed, if the natural sensibility of the aperture of the glottis is not well subdued by previous applications of the nitrate of silver solution, or if the tube, in its introduction, touches roughly the border or lips of the glottis, a spasm of the glottis is certain to follow, which will arrest the further progress of the operation. The epiglottis, which is nearly insensible, (and this you may prove on any person, by thrusting two fingers over the base of the tongue, and touching, or even scratching with the nail, this cartilage,) should be our guide in performing the operation. The strength of the solution for injecting, is from 10 to 25 grains to the ounce of water. Commencing with 10 or 15 grains to the ounce, its strength is subsequently increased, and the amount I now employ is from ½ to 1½ drachms of this solution."*

Allow me further to add, that, latterly, in commencing the injections, I have used a solution still weaker than above denoted. When my patients are prepared for catheterism, by repeated cauterizations of the opening of the glottis and larynx, to reduce the normal sensitiveness of the parts, the tube is then introduced, and a drachm of solution of nitrate of argent., of the strength of from 5 to 10 grains to the ounce of water, is injected through the trachea. Afterwards, the solution may be gradually increased in power; but, at the present day, I seldom employ the remedy, in bronchial injections, of a strength above 20 grains of the salt to an ounce of water.

Should a spasm of the glottis occur, as I have before remarked in this paper, on the insertion of the tube into larynx, the instrument should be promptly withdrawn, and no further attempt be made to proceed with the operation, until the irritation has fully subsided. It is necessary that the applications of the sponge-probang be continued in the intervals of the employment of the tube.

In cases of bronchitis, in asthma, and in early phthisis pulmonalis, even, the use of injections into the bronchi, once or twice a week, operate to diminish the cough, expectoration and dyspnœa, with great certainty, and very many cases of these diseases have recovered under local treatment, after other measures had fail.—*Amer. Med. Monthly.*

---

M. Cloquet has been appointed President of the Academy of Medicine of Paris.

Bailliere of Paris, the well known medical publisher, died recently.

* "Clinical Lectures on Medicine," pp. 608-9.

